# Serum IgA Responses against Pertussis Proteins in Infected and Dutch wP or aP Vaccinated Children: An Additional Role in Pertussis Diagnostics

**DOI:** 10.1371/journal.pone.0027681

**Published:** 2011-11-14

**Authors:** Lotte H. Hendrikx, Kemal Öztürk, Lia G. H. de Rond, Sabine C. de Greeff, Elisabeth A. M. Sanders, Guy A. M. Berbers, Anne-Marie Buisman

**Affiliations:** 1 Centre for Infectious Disease and Control (Clb), National Institute for Public Health and the Environment, Bilthoven, The Netherlands; 2 Research Centre Linnaeus institute, Spaarne Hospital Hoofddorp, Hoofddorp, The Netherlands; 3 Department of Pediatric Immunology and Infectious Diseases, University Medical Centre Utrecht, Utrecht, The Netherlands; Tulane University, United States of America

## Abstract

**Background:**

Whooping cough is a respiratory disease caused by *Bordetella pertussis*, which induces mucosal IgA antibodies that appear to be relevant in protection. Serum IgA responses are measured after pertussis infection and might provide an additional role in pertussis diagnostics. However, the possible interfering role for pertussis vaccinations in the induction of serum IgA antibodies is largely unknown.

**Methods/Principal Findings:**

We compared serum IgA responses in healthy vaccinated children between 1 and 10 years of age with those in children who despite vaccinations recently were infected with *Bordetella pertussis*. All children have been vaccinated at 2, 3, 4 and 11 months of age with either the Dutch whole-cell pertussis (wP) vaccine or an acellular pertussis (aP) vaccine and additionally received an aP booster vaccination at 4 years of age. Serum IgA responses to pertussis toxin (PT), filamentous heamagglutinin (FHA) and pertactin (Prn) were measured with a fluorescent multiplex bead-based immuno-assay. An ELISPOT-assay was used for the detection of IgA-memory B-cells specific to these antigens. Serum IgA levels to all pertussis vaccine antigens were significantly higher in infected children compared with healthy children. High correlations between anti-PT, anti-FHA or anti-Prn IgA and IgG levels were found in infected children and to some degree in wP primed children, but not at all in aP primed children. Highest numbers of IgA-pertussis-specific memory B-cells were observed after infection and generally comparable numbers were found after wP and aP vaccination.

**Conclusions:**

This study provides new insight in the diagnostic role for serum IgA responses against PT in vaccinated children. Since aP vaccines induce high serum IgG levels that interfere with pertussis diagnostics, serum IgA-PT levels will provide an additional diagnostic role. High levels of serum IgA for PT proved specific for recent pertussis infection with reasonable sensitivity, whereas the role for IgA levels against FHA and Prn in diagnosing pertussis remains controversial.

## Introduction

*B.pertussis* establishes infection by attaching to epithelial cells on the human respiratory tract, where toxins are released that cause local and systemic damage [1]. At the mucosal surface, secretory IgA, a dimeric complex, represents the predominant antibody isotype and functions as a first line of defense. The role of serum IgA is less clear. Circulating serum IgA is mainly monomeric and is considered a second line of defense, mediating elimination of pathogens that have breached the mucosal surface [2]. Serum IgA facilitates antibody-dependent cell-mediated cytotoxity and phagocytosis. However, information about the induction of serum IgA and its role after either pertussis vaccination or infection in children is complicated to interpret. Previous studies showed an increase in serum anti-pertussis IgA responses after infection, but not after whole-cell (wP) vaccination in children and adults [3], [4], [5], [6], [7], [8], [9]. In contrast, others demonstrated increased IgA responses to the pertussis vaccine components after acellular pertussis (aP) vaccination in adolescents and adults [10], [11], [12]. However, these serum IgA responses might result from earlier priming by (subclinical) infection. Since the last two decades whooping cough is reemerging in countries with widespread pertussis vaccination coverage, especially in the adolescent and adult population. In most of these countries a wP pertussis vaccine was replaced by an aP vaccine in the 1990s. A serum IgG level against PT above 50–100 IU/ml is currently used for pertussis diagnostics [13]. However, aP vaccines also induce high IgG-PT levels that interfere with pertussis diagnostics. Whether the value of anti-pertussis IgA levels could be indicative for recent infection with *B.pertussis* is a long topic of debate and the possible interfering effects of wP and aP vaccinations on the interpretation of IgA results have not been studied before. Therefore, we evaluated the IgA responses to pertussis toxin (PT), filamentous heamagglutinin (FHA) and pertactin (Prn) in children between 1 and 10 years of age who were vaccinated with either the Dutch wP or aP vaccine at 2, 3, 4 and 11 months of age and additionally received an aP preschool booster vaccination. We compared serum IgA levels in healthy vaccinated children with those in vaccinated children of the same ages who despite previous vaccinations recently were infected with *B.pertussis*.

## Methods

### Ethics Statement

All studies were conducted according to the Declaration of Helsinki, Good Clinical Practice Guidelines with the approval of the relevant ethics review committee (Medisch Ethische Toestingscommissie (METC) in Almere, the Netherlands and the Central Committee on Research Involving Human Subjects (CCMO), the Hague, the Netherlands). Written informed consent was obtained from parents or legal representatives before the start of the study.

### Study population

**Figure 1:**
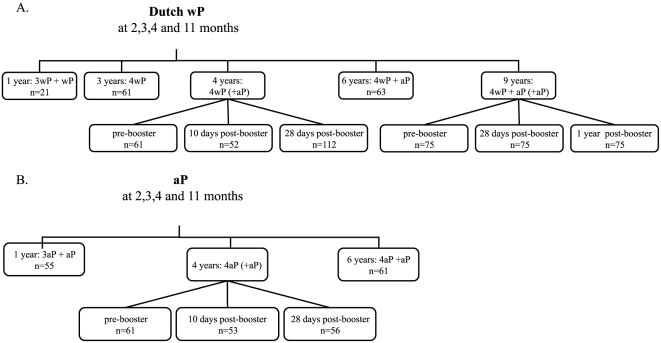
The number of healthy children per age group. (A) children primed with the Dutch wP vaccine and (B) children primed with aP vaccines.

Healthy children of different ages (12 months, 3, 4, 6 and 9 years) included in this study originated from three different cohort studies; (1) 51 children 12 months of age from whom one blood sample was taken at one month after the routine 4^th^ vaccination at 11 months (ISRCTN97785537); (2) 580 children of 3, 4 or 6 years of age with one blood sample at the presented ages or children aged 4 years with one blood sample either before or 10 days or 28 days after the preschool booster vaccination (ISRCTN65428640) [14]; 75 children 9 years of age with sequential blood samples before, one month and one year after a second aP booster vaccination at 9 years of age (ISRCTN64117538) (figure 1). In addition, a fourth cohort consisted of 73 children 1 to 10 years of age who were vaccinated according to the national immunization program (NIP, see below) but in whom a recent pertussis infection was diagnosed [15]. Recent pertussis infection was defined as having a high single serum anti-IgG-PT level (>100 U/ml) or a positive PCR confirmation. One blood sample was taken per child on average 42 days (range 3–119 days) after the first symptoms of whooping cough.

### Vaccinations

All healthy and infected children had received either the Dutch whole-cell pertussis vaccine (DTwP-IPV- Hib, NVI, Bilthoven, the Netherlands) or an acellular vaccine (DTaP-IPV- Hib, *Infanrix-IPV-Hib*™, GlaxoSmithKline Biologicals S.A., Rixensart, Belgium) at 2, 3, 4 and 11 months of age according to the NIP. The Dutch wP vaccine was replaced by an aP vaccine in 2005. Therefore, subgroups of children of 12 months of age had received either 4 injections of wP or 4 of aP vaccines at 2, 3, 4 and 11 months of age and a third subgroup had received 3 wP vaccinations at 2, 3 and 4 months of age and one aP vaccination at 11 months of age. In children 4 years and older, an additional preschool booster vaccination with either ACV-SB or *Infanrix*™ (both from GSK) or with *Triaxis*™ (Sanofi Pasteur, Lille, France) was administered at 4 years of age. ACV-SB and *Infanrix*™ both contained the same amounts of pertussis antigens (high-dose); 25 µg of PT, 25 µg of FHA and 8 µg of Prn, whereas *Triaxis*™ contained 2.5 µg of PT, 5 µg of FHA, 3 µg of Prn and 5 µg of combined fimbriae type 2 and 3 (Fim2/3) (low-dose) [14].

Children 9 years of age had received a second aP booster vaccination, *Boostrix-IPV*™ (GSK), that contained 8 µg of PT, 8 µg of FHA and 2.5 µg of Prn. Of the 73 children who had been infected with *B.pertussis*, 71 children had been vaccinated according to the NIP (63 wP primed children and 8 aP primed children) and 2 children (7 and 8 years of age) had not received any pertussis vaccination before infection.

### Serological assays

For measurement of plasma IgA levels directed against the 3 *B. pertussis* vaccine antigens (PT, FHA and Prn) the fluorescent bead-based multiplex immunoassay (MIA) was used as previously described [14], [16] with some modifications. To prevent possible inter-immunoglobulin isotype competition, plasma samples were depleted of IgG by adding GullSORB (10∶1 vol/vol) (meridian Bioscience Inc., Cincinnati, OH). Moreover, 50 µl of a 1/100 dilution of Goat F(ab′)2-anti-Human IgA was added to each well for 30 minutes before adding 50 µl R-Phycoerythrin conjugated Goat anti-Mouse IgA (Southern Biotechnologies, Alabama). The in-house reference serum was calibrated against the human reference pertussis antiserum lot 3 for PT and FHA and lot 4 for Prn (CBER, FDA). Next to in-house controls in a single dilution, a 3-fold serial dilution of the reference serum was added over 8 wells on each plate. Serum IgA antibodies against a crude cell-membrane preparation of *B.pertussis* were previously measured with an in-house ELISA as described earlier [15].

### B-cell isolation, stimulation and ELISPOT-assays

In 149 randomly selected healthy and infected children between 3 and 10 years of age (on average 10 per group, range 5–17), we performed total IgA and at least one pertussis antigen-specific enzyme-linked immunospot (ELISPOT)-assays as previously described [17], [18]. Plates were coated with 10 µg/ml anti-human IgA-Fc-specific and incubated the plate with alkaline phosphatase conjugated goat anti-human IgA for the detection of IgA-specific memory B-cells to the pertussis antigens [17], [18]. Mean spot values of the non-coated wells were used as negative controls. From all mean spot values of the antigen-coated wells per sample, the negative controls were subtracted. The numbers of antigen-specific IgA-memory B-cells were presented per 10^5^ B-cells. Due to low numbers of IgA antigen-specific memory B-cells, we presented the percentage of children with at least 1 antigen-specific memory B-cell and defined this as a positive memory B-cell response.

### Statistical methods

Anti-PT, anti-FHA and anti-Prn IgA levels were expressed as geometric mean concentrations (GMCs) with a 95% confidence interval (CI). A positive IgA response was defined as an IgA level above 1 EU/ml for PT, FHA and Prn. The Mann-Whitney-U-test was used for comparison between the different groups. To analyze the correlation between variables Spearman correlations (r_s_) and linear regression analysis was used. P<0.05 was considered significantly different.

## Results

### IgA levels after wP or aP vaccinations or pertussis infection

**Figure 2:**
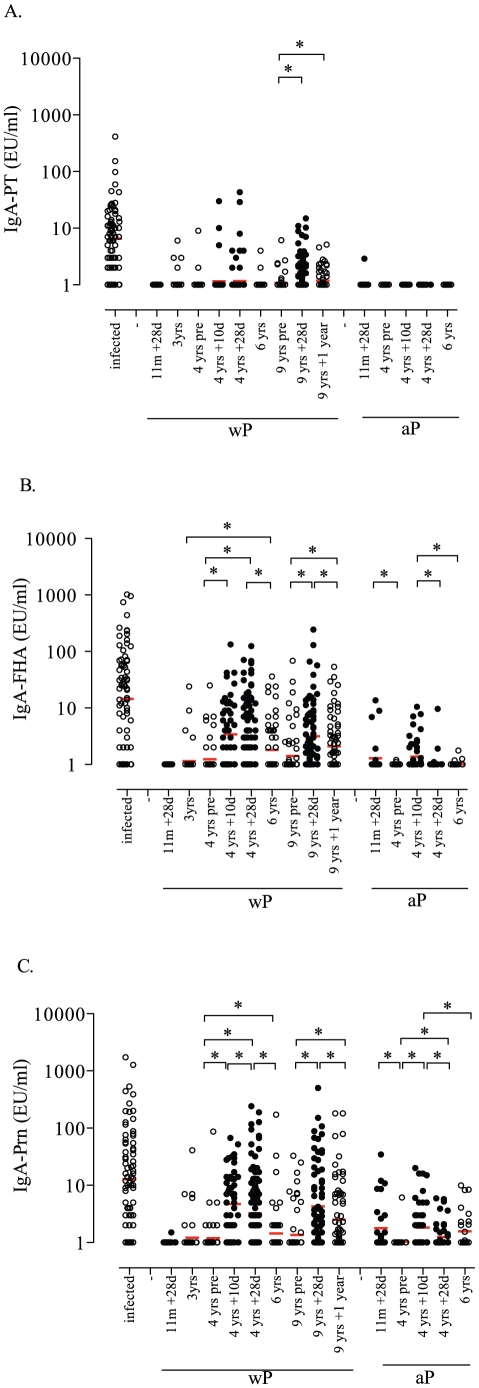
IgA levels. IgA responses specific for PT (A), FHA (B) and Prn (C) for each child in the on the *x*-axis presented group of children. The red horizontal bars indicate the GMC. The 10 and 28 days post-booster responses are indicated by the black circles and all other groups are indicate by open circles. * significant differences (p<0.05) are indicated for the healthy children between the presented age groups in similar primed children. Differences between infected children and the various groups of healthy wP and aP primed children are described in paragraph 3.1 of the results section.

Infected children showed significant higher IgA responses to PT, FHA and Prn than both Dutch wP and aP primed healthy children at 1, 3, 4, 6 and 9 years of age (figure 2). In addition, a positive PT-, FHA- and Prn-specific IgA response was found in 84%, 78% and 80% of the infected children respectively, whereas 6%, 58% and 75% of the wP primed children aged 4 years and 0%, 24% and 44% of the aP primed children aged 4 years showed a positive IgA response at day 10 after the preschool booster vaccination, respectively (data not shown). IgA responses to all 3 antigens were positive in 62% of the infected children, but only 9% of all wP primed children (pre- and post-booster) and in 0% of all aP primed children. In wP primed children at 4 years of age significantly higher post-booster FHA- and Prn-responses were observed and at 6 years of age significantly higher FHA-responses were shown compared with aP primed children of the same ages (figure 2). Five years after the preschool booster at 4 years of age in wP primed children of 9 years of age, anti-pertussis IgA responses had returned to pre-booster levels. However, all pertussis-specific IgA responses significantly increased after a second booster vaccination at 9 years of age and after one-year follow-up anti-FHA and anti-Prn IgA levels still significantly exceeded pre-booster levels.

### The effect of the time of blood sampling on IgA responses

In infected children that were older than 4 years significantly higher IgA responses to PT and FHA were observed compared with children younger than 4 years, at least in those children where blood sampling had occured within 30–60 days after the start of clinical symptoms of whooping cough (table 1). After 60 days, GMCs of anti-PT, anti-FHA and anti-Prn IgA levels were comparable in children above 4 years of age compared with those younger than 4 years.

**Table 1:** The effect of the time of blood sampling since the start of pertussis symptoms on serum IgA responses to PT, FHA and Prn in children younger and older than 4 years of age.

			IgA-PT		IgA-FHA		IgA-Prn	
Children <4 years		n =	GMC	95% CI	GMC	95% CI	GMC	95% CI
Timepoint	<30 days	13	4.3	2.3–8.2	7.1	2.9–17.5	13.5	3.2–57.0
	30–60 days	17	7.0	4.2–11.8	14.5	5.5–38.4	15.2	4.6–50.3
	>60 days	9	3.6	1.2–11.1	18.8	1.1–336.2	16.5	0.9–310.5

Children >4 years		n =	GMC	95% CI	GMC	95% CI	GMC	95% CI
Timepoint	<30 days	13	11.5	4.4–29.7	16.6	5.0–55.4	11.7	5.5–25.0
	30–60 days	11	14.8* #	8.9–24.4	93.8∫ #	41.1–214.1	18.3	8.4–39.7
	>60 days	12	3.4	1.1–10.4	14.6	1.3–163.7	21.5	3.3–139.2

*significantly higher as compared to >60 days GMC.

∫significantly higher as compared to <30 days GMC.

# significantly higher as compared to GMC at same time point in children <4 years of age.

### Correlation between IgA and IgG levels

**Figure 3:**
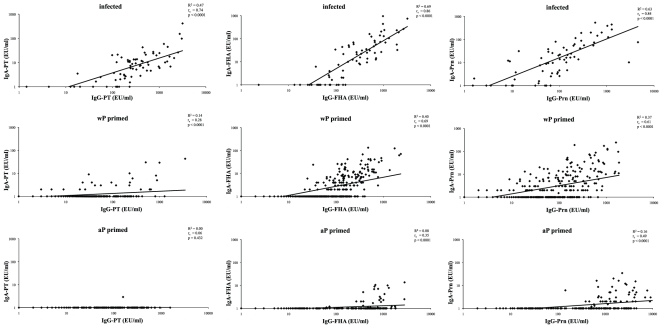
Correlations between serum IgA and IgG responses. Spearman correlations and linear regression analysis between serum IgA and serum IgG responses specific for PT, FHA and Prn presented for infected (A), wP (B) and aP (C) primed children.

High correlations between anti-PT, anti-FHA and anti-Prn IgA and IgG levels were found in infected children and to some degree in Dutch wP primed children, but not in aP primed children (figure 3). For FHA and Prn, highest correlations were observed in infected and wP primed children, while for PT only a high correlation between IgA and IgG levels was found in infected children. Low correlations for FHA and Prn were found in aP primed children.

**Figure 4:**
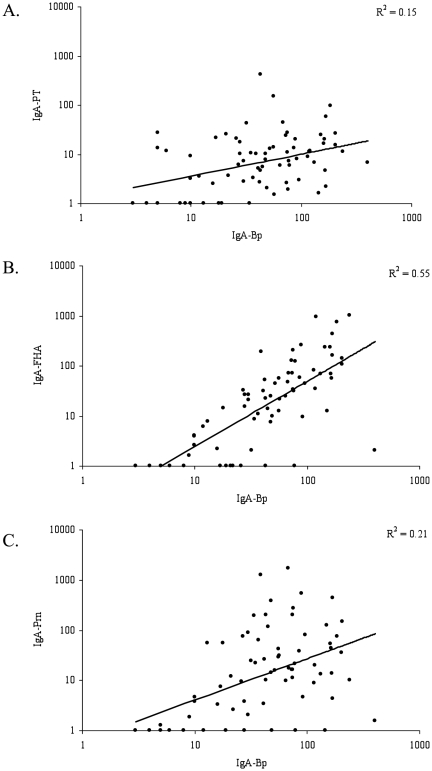
Correlations between serum IgA-Bp and IgA-PT, -FHA and -Prn in infected children. Serum IgA-Bp levels are presented on the *x*-axis and serum IgA-PT (A), -FHA (B) and -Prn (C) on the *y*-axis.

As IgA levels against pertussis crude cell-membranes (IgA-Bp) were used for pertussis diagnostics in several countries, we correlated these levels with our pertussis vaccine antigen-specific IgA levels in infected children. No correlation between serum IgA-Bp antibodies and serum IgA levels against PT was found, while IgA-Bp levels correlated with anti-FHA IgA levels and to a lesser extent with serum anti-Prn levels (figure 4).

### Pertussis-specific IgA memory B-cells responses

The percentage of total IgA-producing memory B-cells in infected children (on average 6.8%) was significantly higher than in the healthy children (on average 3.0%). Also, the number of PT-, FHA- and Prn-specific memory B-cells was significantly higher in infected children as compared to healthy children. Between the healthy Dutch wP and aP primed children of different ages, no differences in percentages of total IgA-producing B-cells and no differences in the number of PT-, FHA- and Prn-specific memory B-cell responses were found (data not shown). In general, about 50% of the children showed a positive PT-, FHA- or Prn-specific memory B-cell response (table 2). The majority of the Dutch wP primed children at 9 years of age showed PT-, FHA- and Prn-specific memory B-cells 28 days after the second booster vaccination. Remarkably, 4/5 of the aP primed children at 4 years of age showed already FHA-specific memory B-cells even before the preschool booster vaccination.

**Table 2:** Percentages (%) of children with a positive PT-, FHA- and Prn- specific IgA- memory B-cell response in the infected and healthy Dutch wP primed and aP primed children of different ages (number of children per group).

	Infected	Dutch wP at 2, 3, 4 and 11 months								aP at 2, 3, 4 and 11 months			
age	1–10	3	4	4	4	6	9	9	9	4	4	4	6
post-booster	-	-	-	10 days	28 days	-	-	28 days	1 year	-	10 days	28 days	-
PT B-cells	70% (10)	42% (12)	62% (13)	38% (8)	45% (11)	45% (11)	58% (12)	63% (8)	60% (10)	43% (7)	50% (10)	29% (17)	21% (14)
FHA B-cells	70% (10)	22% (9)	54% (13)	17% (6)	29% (7)	50% (12)	33% (6)	60% (5)	40% (5)	80% (5)	13% (8)	33% (12)	50% (14)
Prn B-cells	80% (10)	77% (13)	71% (14)	44% (9)	73% (11)	50% (12)	50% (12)	75% (8)	50% (10)	50% (10)	55% (11)	60% (15)	21% (14)

## Discussion

In this study we showed an increased serum IgA level against PT in vaccinated children who had recently been infected with *B.pertussis*, but not in healthy aP vaccinated children and only in a few Dutch wP vaccinated children. Remarkably, the high correlation between serum IgA and IgG anti-PT antibodies in infected children, but not in the healthy wP and aP vaccinated children, strengthens the role of serum PT-specific IgA as a diagnostic parameter for pertussis also in the vaccinated population. We found that anti-PT IgA levels in serum had a high specificity and a reasonable sensitivity. Since aP vaccinations induce high levels of IgG antibodies against PT, the presence of serum IgA-PT responses will help to differentiate between vaccine induced antibody levels and those induced by natural infection. Only 6% of the Dutch wP primed children showed a positive IgA-response for PT shortly after the preschool booster with an aP vaccine. Interestingly, this percentage is concurrent with the sero-prevalence of pertussis in children in the Netherlands at the time the study was conducted [19]. In these children who might have been primed by previous exposure to *B.pertussis* leading to (subclinical) infection, serum IgA-PT levels could consequently have increased again after re-stimulation by booster vaccination. The overall absence of anti-PT antibodies in aP primed children suggests that infection has not occurred in these children. This implicitly demonstrates that the implementation of the aP vaccine for infant immunization has led to improved protection against whooping cough in young children in the Netherlands as previous data already suggested [19], [20]. The absence of a dose-response effect for IgA levels after pertussis booster vaccination with low or high pertussis antigen content in both Dutch wP and aP primed healthy children at 4 years of age actually emphasizes that priming for pertussis-specific IgA responses is induced by infection rather than by vaccination. Moreover, as 6% of the population is suggested to be annually infected with *B.pertussis* [19], about half of our wP primed children 9 years of age should show an elevated serum IgA-PT level at one month after the booster as is confirmed by our data (32/75 children 9 years of age).

The role for IgA levels against the other pertussis antigens FHA and Prn in pertussis diagnostics remains controversial. Serum IgA responses against FHA and Prn were not limited to the infected children only, but were also observed in healthy Dutch wP primed and to a lesser extent in healthy aP primed children. Moreover, serum IgA responses against pertussis crude cell-membranes also lack specificity and correlated with the *B.pertussis* non specific anti-FHA levels, but only marginally with the *B.pertussis* specific anti-PT levels. Since FHA and Prn are adhesion factors of *B.pertussis*, these antibodies might illustrate the ongoing circulation of *B.pertussis*. Moreover, while PT is a toxin unique to *B.pertussis*, Prn antigen also belongs to other *Bordetellae* species [21] and other organisms also induce FHA antibodies [22], [23]. As our previous studies already indicated [14], [18] the Prn component in the aP vaccine seemed to be highly immunogenic resulting in extremely high IgG-Prn levels after aP vaccination. This high immunogenicity may also explain the small increase in IgA-Prn levels observed in several children in the aP vaccinated groups of 12 months and 6 years of age. The GMCs of the PT, FHA and Prn IgA antibodies in the infected children are in agreement with those previously measured in *B.pertussis* confirmed infected adults with unknown pertussis vaccination-history [7], [24]. Moreover, a small increase in anti-PT IgA levels and a higher increase in anti-FHA and anti-Prn IgA levels after an aP booster vaccination have also been observed in some adolescents and adults [10], [11], [12] and are in line with our findings in children.

It was previously suggested that serum IgA-PT antibodies represent recent infection, but only 50% of children with pertussis actually showed an IgA-PT response [25]. In an Australian study the sensitivities of whole-cell, PT, FHA or Prn IgA assays varied between 24% and 64% [4]. In our study, however, we found 80% sensitivity of serum anti-PT IgA levels in pertussis infected children that proved independent of the time of blood sampling. As the MIA is a highly accurate and sensitive assay, even in the lower limit of quantification, the minimal defined level of 1 EU/ml for a positive IgA immune response is justified. Future monitoring of serum IgA antibodies in the young aP primed population will be useful to further corroborate the role of serum IgA antibodies in single serum diagnostics for pertussis.

Surprisingly, despite lower serum anti-pertussis IgA levels in healthy aP primed children compared with healthy wP primed children, comparable numbers of children from both groups showed detectable circulating pertussis-specific IgA memory B-cells. Antigen-specific IgA memory B-cells are derived in the mucosa associated lymphoid tissue and migrate via the blood to the secretory effector sites, e.g. the oral mucosa where sIgA is locally produced after antigen challenge [26]. This might explain why both wP and aP vaccinated children have comparable numbers of pertussis-specific IgA memory B-cells, since the contact with *B.pertussis* will be similar for both groups due to the high circulation. However, in the wP vaccinated group higher serum IgA responses were observed as compared to aP vaccinated children, probably because infection with *B.pertussis* occurs more frequently in this group. Moreover, we have previously shown that some years after vaccination pertussis specific IgG memory B-cells do not perse correlate with serum IgG levels [18]. In addition, long-lived plasmacells home to the bone marrow and locally produce background serum IgA levels [2]. However, it is unknown whether the serum IgA levels are either induced by circulating antigens or polyclonal stimulation or are a result of leakage from the mucosal surfaces into the circulation [26]. Only one study from 20 years ago showed a correlation for FHA and PT between serum IgA and IgA levels in saliva of pertussis-infected individuals. The diagnostic value of salivary IgA seemed rather weak for pertussis [27], whereas circulating pathogen-specific IgA antibodies might reflect those at the mucosal surfaces as demonstrated for rotavirus and poliovirus [28], [29], [30]. In general, mucosal IgA responses are important in the protection against pathogens as found in newborns and patients with IgA deficiency [31], [32]. However, the role for serum IgA in protection against whooping cough remains unresolved. Murine studies showed that IgA exhibits bactericidal effector functions [33], whereas IgA was not required to clear *B.pertussis* [34]. In a recent murine study a superior protection and promising long-term immune response was found after a single intranasal dose of a live-attenuated *B.pertussis* vaccine, named BPZE1, compared to parenterally administred aP vaccines [35]. This suggest an important role for mucosal immune responses in protection against pertussis infection. In our study we found no serum IgA responses after aP vaccinations, whereas protection against pertussis has improved after implementation of these aP vaccines. Therefore, serum IgA antibodies do not seem to be essential for pertussis protection after parentarelly administered vaccines. However, after infection this situation might be different and it is very likely that IgA antibodies play an important role in the clearance of the infection at the mucosa. It will be important to further elucidate anti-PT IgA levels in aP primed individuals who have been infected, to prove the role for serum anti-PT IgA levels in diagnostics. Moreover, the effect of different pertussis vaccinations as well as natural infection on salivary IgA will be important to further elucidate the role of mucosal IgA in protection against whooping cough [31], [32], [33], [34], [35].

In conclusion, we demonstrated that a high specificity of serum IgA levels against PT with a reasonable sensitivity recent pertussis infection in Dutch wP and aP vaccinated children. While infection induced anti-PT IgA antibodies, pertussis vaccines clearly did not. Especially in the aP vaccinated population, serum IgA-PT responses will be of great value for the diagnostics of pertussis. A better understanding of serum and mucosal IgA responses and their role in protection against whooping cough should finally result in a further improvement of the pertussis vaccines for infants.

## References

[1] Parton R (1999). Review of the biology of Bordetella pertussis.. Biologicals.

[2] Woof JM, Kerr MA (2006). The function of immunoglobulin A in immunity.. J Pathol.

[3] Nagel J, Poot-Scholtens EJ (1983). Serum IgA antibody to Bordetella pertussis as an indicator of infection.. J Med Microbiol.

[4] Poynten M, Hanlon M, Irwig L, Gilbert GL (2002). Serological diagnosis of pertussis: evaluation of IgA against whole cell and specific Bordetella pertussis antigens as markers of recent infection.. Epidemiol Infect.

[5] Novotny P, Macaulay ME, Hart TC, Skvaril F (1991). Analysis of antibody profiles in children with whooping cough.. Dev Biol Stand.

[6] Heininger U, Cherry JD, Stehr K (2004). Serologic response and antibody-titer decay in adults with pertussis.. Clin Infect Dis.

[7] Deen JL, Mink CA, Cherry JD, Christenson PD, Pineda EF (1995). Household contact study of Bordetella pertussis infections.. Clin Infect Dis.

[8] Bonhoeffer J, Bar G, Riffelmann M, Soler M, Heininger U (2005). The role of Bordetella infections in patients with acute exacerbation of chronic bronchitis.. Infection.

[9] Thomas MG, Ashworth LA, Miller E, Lambert HP (1989). Serum IgG, IgA, and IgM responses to pertussis toxin, filamentous hemagglutinin, and agglutinogens 2 and 3 after infection with Bordetella pertussis and immunization with whole-cell pertussis vaccine.. J Infect Dis.

[10] Le T, Cherry JD, Chang SJ, Knoll MD, Lee ML (2004). Immune responses and antibody decay after immunization of adolescents and adults with an acellular pertussis vaccine: the APERT Study.. J Infect Dis.

[11] Littmann M, Hulsse C, Riffelmann M, Wirsing von Konig CH (2008). Long-term immunogenicity of a single dose of acellular pertussis vaccine in paediatric health-care workers.. Vaccine.

[12] Knuf M, Zepp F, Meyer C, Grzegowski E, Wolter J (2006). Immunogenicity of a single dose of reduced-antigen acellular pertussis vaccine in a non-vaccinated adolescent population.. Vaccine.

[13] Guiso N, Berbers G, Fry NK, He Q, Riffelmann M (2010). What to do and what not to do in serological diagnosis of pertussis: recommendations from EU reference laboratories.. Eur J Clin Microbiol Infect Dis.

[14] Hendrikx LH, Berbers GA, Veenhoven RH, Sanders EA, Buisman AM (2009). IgG responses after booster vaccination with different pertussis vaccines in Dutch children 4 years of age: effect of vaccine antigen content.. Vaccine.

[15] de Greeff SC, Mooi FR, Westerhof A, Verbakel JM, Peeters MF (2010). Pertussis disease burden in the household: how to protect young infants.. Clin Infect Dis.

[16] van Gageldonk PG, van Schaijk FG, van der Klis FR, Berbers GA (2008). Development and validation of a multiplex immunoassay for the simultaneous determination of serum antibodies to Bordetella pertussis, diphtheria and tetanus.. J Immunol Methods.

[17] Buisman AM, de Rond CG, Ozturk K, Ten Hulscher HI, van Binnendijk RS (2009). Long-term presence of memory B-cells specific for different vaccine components.. Vaccine.

[18] Hendrikx LH, Ozturk K, de Rond LG, Veenhoven RH, Sanders EA (2011). Identifying long-term memory B-cells in vaccinated children despite waning antibody levels specific for Bordetella pertussis proteins.. Vaccine.

[19] de Greeff SC, de Melker HE, van Gageldonk PG, Schellekens JF, van der Klis FR (2010). Seroprevalence of Pertussis in the Netherlands: Evidence for Increased Circulation of Bordetella pertussis.. PLoS One.

[20] de Greeff SC, Mooi FR, Schellekens JF, de Melker HE (2008). Impact of acellular pertussis preschool booster vaccination on disease burden of pertussis in The Netherlands.. Pediatr Infect Dis J.

[21] Inatsuka CS, Xu Q, Vujkovic-Cvijin I, Wong S, Stibitz S (2010). Pertactin is required for Bordetella species to resist neutrophil-mediated clearance.. Infect Immun.

[22] Vincent JM, Cherry JD, Nauschuetz WF, Lipton A, Ono CM (2000). Prolonged afebrile nonproductive cough illnesses in American soldiers in Korea: a serological search for causation.. Clin Infect Dis.

[23] Isacson J, Trollfors B, Taranger J, Lagergard T (1995). Acquisition of IgG serum antibodies against two Bordetella antigens (filamentous hemagglutinin and pertactin) in children with no symptoms of pertussis.. Pediatr Infect Dis J.

[24] Cherry JD, Beer T, Chartrand SA, DeVille J, Beer E (1995). Comparison of values of antibody to Bordetella pertussis antigens in young German and American men.. Clin Infect Dis.

[25] von Linstow ML, Pontoppidan PL, von Konig CH, Cherry JD, Hogh B (2010). Evidence of Bordetella pertussis infection in vaccinated 1-year-old Danish children.. Eur J Pediatr.

[26] Brandtzaeg P (2007). Do salivary antibodies reliably reflect both mucosal and systemic immunity?. Ann N Y Acad Sci.

[27] Zackrisson G, Lagergard T, Trollfors B, Krantz I (1990). Immunoglobulin A antibodies to pertussis toxin and filamentous hemagglutinin in saliva from patients with pertussis.. J Clin Microbiol.

[28] Franco MA, Angel J, Greenberg HB (2006). Immunity and correlates of protection for rotavirus vaccines.. Vaccine.

[29] Gonzalez R, Franco M, Sarmiento L, Romero M, Schael IP (2005). Serum IgA levels induced by rotavirus natural infection, but not following immunization with the RRV-TV vaccine (Rotashield), correlate with protection.. J Med Virol.

[30] Buisman AM, Abbink F, Schepp RM, Sonsma JA, Herremans T (2008). Preexisting poliovirus-specific IgA in the circulation correlates with protection against virus excretion in the elderly.. J Infect Dis.

[31] Hanson LA, Korotkova M (2002). The role of breastfeeding in prevention of neonatal infection.. Semin Neonatol.

[32] Brandtzaeg P (1995). The role of humoral mucosal immunity in the induction and maintenance of chronic airway infections.. Am J Respir Crit Care Med.

[33] Hellwig SM, van Spriel AB, Schellekens JF, Mooi FR, van de Winkel JG (2001). Immunoglobulin A-mediated protection against Bordetella pertussis infection.. Infect Immun.

[34] Wolfe DN, Kirimanjeswara GS, Goebel EM, Harvill ET (2007). Comparative role of immunoglobulin A in protective immunity against the Bordetellae.. Infect Immun.

[35] Feunou PF, Kammoun H, Debrie AS, Mielcarek N, Locht C (2010). Long-term immunity against pertussis induced by a single nasal administration of live attenuated B. pertussis BPZE1.. Vaccine.

